# Tuberculosis Prevalence and Predictors Among Health Care-Seeking People Screened for Cough of Any Duration in Ethiopia: A Multicenter Cross-Sectional Study

**DOI:** 10.3389/fpubh.2021.805726

**Published:** 2022-02-25

**Authors:** Hussen Mohammed, Lemessa Oljira, Kedir Teji Roba, Esther Ngadaya, Tsegahun Manyazewal, Tigest Ajeme, Nicholaus P. Mnyambwa, Abebaw Fekadu, Getnet Yimer

**Affiliations:** ^1^Department of Public Health, College of Medicine and Health Sciences, Dire Dawa University, Dire Dawa, Ethiopia; ^2^Centre for Innovative Drug Development and Therapeutic Trials for Africa (CDT-Africa), College of Health Sciences, Addis Ababa University, Addis Ababa, Ethiopia; ^3^School of Public Health, College of Health and Medical Sciences, Haramaya University, Harar, Ethiopia; ^4^School of Nursing and Midwifery, College of Health and Medical Sciences, Haramaya University, Harar, Ethiopia; ^5^Muhimbili Research Centre, National Institute for Medical Research, Dar es Salaam, Tanzania; ^6^Global Health and Infection Department, Brighton and Sussex Medical School, Brighton, United Kingdom; ^7^Ohio State Global One Health Initiative, Office of International Affairs, The Ohio State University, Addis Ababa, Ethiopia

**Keywords:** prevalence, cough, tuberculosis, chest X-ray, algorithm, Ethiopia

## Abstract

**Background:**

Tuberculosis (TB) remains a major cause of morbidity and mortality in sub-Saharan Africa. This high burden is mainly attributed to low case detection and delayed diagnosis. We aimed to determine the prevalence and predictors of TB among health care-seeking people screened for cough of any duration in Ethiopia.

**Methods:**

In this multicenter cross-sectional study, we screened 195,713 (81.2%) for cough of any duration. We recruited a sample of 1,853 presumptive TB (PTB) cases and assigned them into three groups: group I with cough ≥2 weeks, group II with cough of <2 weeks, and group III pregnant women, patients on antiretroviral therapy, and patients with diabetes. The first two groups underwent chest radiograph (CXR) followed by sputum Xpert MTB/RIF assay or smear microscopy. The third group was exempted from CXR but underwent sputum Xpert MTB/RIF assay or smear microscopy. TB prevalence was calculated across the groups and TB predictors were analyzed using modified Poisson regression to compute adjusted prevalence ratio (aPR) with a 95% confidence interval (CI).

**Results:**

The overall prevalence of PTB was 16.7% (309/1853). Of the positive cases, 81.2% (251/309) were in group I (cough ≥2 weeks), 14.2% (44/309) in group II (cough of <2), and 4.5% (14/309) in group III (CXR exempted). PTB predictors were age group of 25–34 [aPR = 2.0 (95% CI 1.3–2.8)], history of weight loss [aPR = 1.2 (95% CI 1.1–1.3)], and TB suggestive CXRs [aPR = 41.1 (95% CI 23.2–72.8)].

**Conclusion:**

The prevalence of confirmed PTB among routine outpatients was high, and this included those with a low duration of cough who can serve as a source of infection. Screening all patients at outpatient departments who passively report any cough irrespective of duration is important to increase TB case finding and reduce TB transmission and mortality.

## Introduction

Prevalence, incidence, and death rates from TB are steadily declining globally (1) because of improved diagnosis and treatment, however, this is not evident in most countries in sub-Saharan Africa (SSA) where low case detection due to missed diagnoses or delayed diagnoses, drug risistance and problems with access to high-quality care lead to a higher risk of death, suffering, and catastrophic financial consequences (1). Although it is well-recognized that early TB diagnosis and prompt treatment is a cornerstone to its control, early case detection and timely treatment also reduce morbidity and mortality associated with TB. TB has become a health priority in SSA where the lack of modern facilities for proper diagnosis and management has left a majority of the patients undiagnosed and continue to spread the disease (2, 3). In most of SSA, TB case finding is through passive case finding and where possible, provider-initiated active case finding of symptomatic people, in a predetermined target group such as HIV infected individuals (4).

Under passive case finding an individual is required to report to a health facility for care. In Kenya, Tanzania, Sudan, Uganda, and Ethiopia, for an individual to be recognized as a presumptive TB patient, they need to report to a health facility with a cough of two or more weeks with or without accompanying symptoms (5, 6), limiting the opportunity to capture those who report less duration of a cough, and women and children attending reproductive and child health (RCH) clinics. In these countries, screening for TB has largely been integrated to outpatients department (OPD) and HIV/AIDs clinics (5, 6) but is challenged by high patient load, shortage of manpower and financing, and interruption of laboratory supplies (5, 6). To a large extent, passive case finding depends on individual self-initiative to visit a healthcare facility and report a cough with proper duration, and on a degree of alertness of health workers to identify a patient (7).

In Ethiopia, TB case detection is below the World Health Organization (WHO) target. In 2019, 29.3% of cases were not notified to the national TB program (NTP) (1). It is known that women are good attendants of RCH clinics either for their reproductive health services or the health of their children. Besides, although chest X-ray (CXR) has been recently promoted and recommended by the WHO as a useful tool for TB screening and triaging algorithms (8), it has not been implemented in Ethiopia except for smear-negative patients after 2 weeks of antibiotics (9). CXR has been reported to be the most sensitive TB screening tool (with very low specificity though) since a significant proportion of TB patients are asymptomatic (10). When used to triage who should be tested with GeneXpert, CXR has been reported to reduce the number of individuals tested and, thus, reduce the risk of overdiagnosis. Given the high costs associated with GeneXpert, this improves the efficiency of GeneXpert (11).

Evidence from recent studies for interventions to increase TB case detection includes the following: (1) in Tanzania, detection of smear-positive TB among outpatient department attendees who reported a cough of <2 weeks was as high as for those who reported cough of 2 weeks or more (12). (2) Ngadaya et al. (12, 13) reported that the prevalence of smear-positive TB among women with a cough attending RCH was 3.8% (13). (3) Screening people who have cough <2 weeks can increase the number of early-diagnosed pulmonary TB cases (14). (4) A study in Ethiopia on OPD attendees with a cough of any duration revealed TB cases among people with a cough of <2 weeks (15). (5) Systematic screening of targeted groups has been feasible and improved TB case detection in low-resource settings (16).

In this study, we aimed to determine the prevalence and predictors of TB among health care-seeking people screened for cough of any duration in Ethiopia.

## Methods

### Settings and Populations

We conducted implementation research with a cross-sectional study at four health facilities. Hiwot Fana Specialized University Hospital and Zewditu Memorial Hospital are found in urban settings. Two Chelenko Primary Hospital and Melka Jebdu Health Center are found in rural settings. In short, the facilities were selected by stratified random sampling based on the settings (urban and rural). The study populations who screened were people who sought health care at the study health facilities' units of outpatient departments (OPDs), reproductive and child health clinics, antiretroviral therapy, pregnant women, and diabetes mellitus (DM) clinics during the study period. The study was conducted from July 2019 to March 2020 and from August 2020 to December 2020. The study was interrupted because of the COVID-19 pandemic from April 2020 to July 2020 (17–19).

### Sample Size Determination and Sampling Techniques

A sample size calculation was done with Epi info version 6 with the assumption of 14.7% prevalence of pulmonary TB among individuals who presented with cough of 2 weeks or more and 9.9% prevalence of pulmonary TB among individuals who presented with cough of <2 weeks (20), with 95% confidence interval, and 80% power. Using these assumptions, the minimum calculated sample size was 1550. By adding a 15% non-response rate, the total sample size was 1783. We recruited 1,853 presumptive TB cases and included all.

### Case Definitions

Presumptive TB cases: patients with cough of ≥2 weeks with any chest X-ray results, cough <2 weeks with chest X-ray abnormality suggestive of TB, and cough of any duration for pregnant women, ART, and diabetic patients with or without the presence of night sweats, fever, hemoptysis or loss of weight were presumptive TB cases who were eligible to be evaluated (9). Pulmonary tuberculosis (PTB): a participant with lung TB confirmed by Xpert/smear microscopy or clinically diagnosed as per Ethiopian national TB guidelines (9) and the WHO (21). Bacteriologically-confirmed TB case: a patient from whom at least one sputum was positive for mycobacterium TB either by Xpert/smear microscopy (9, 21). Clinically diagnosed TB case: a participant who did not fulfill the criteria for a bacteriologically-confirmed case, but was diagnosed with TB by an experienced clinician and given a full course of TB treatment (9, 21).

### Screening Procedures

**Figure 1:**
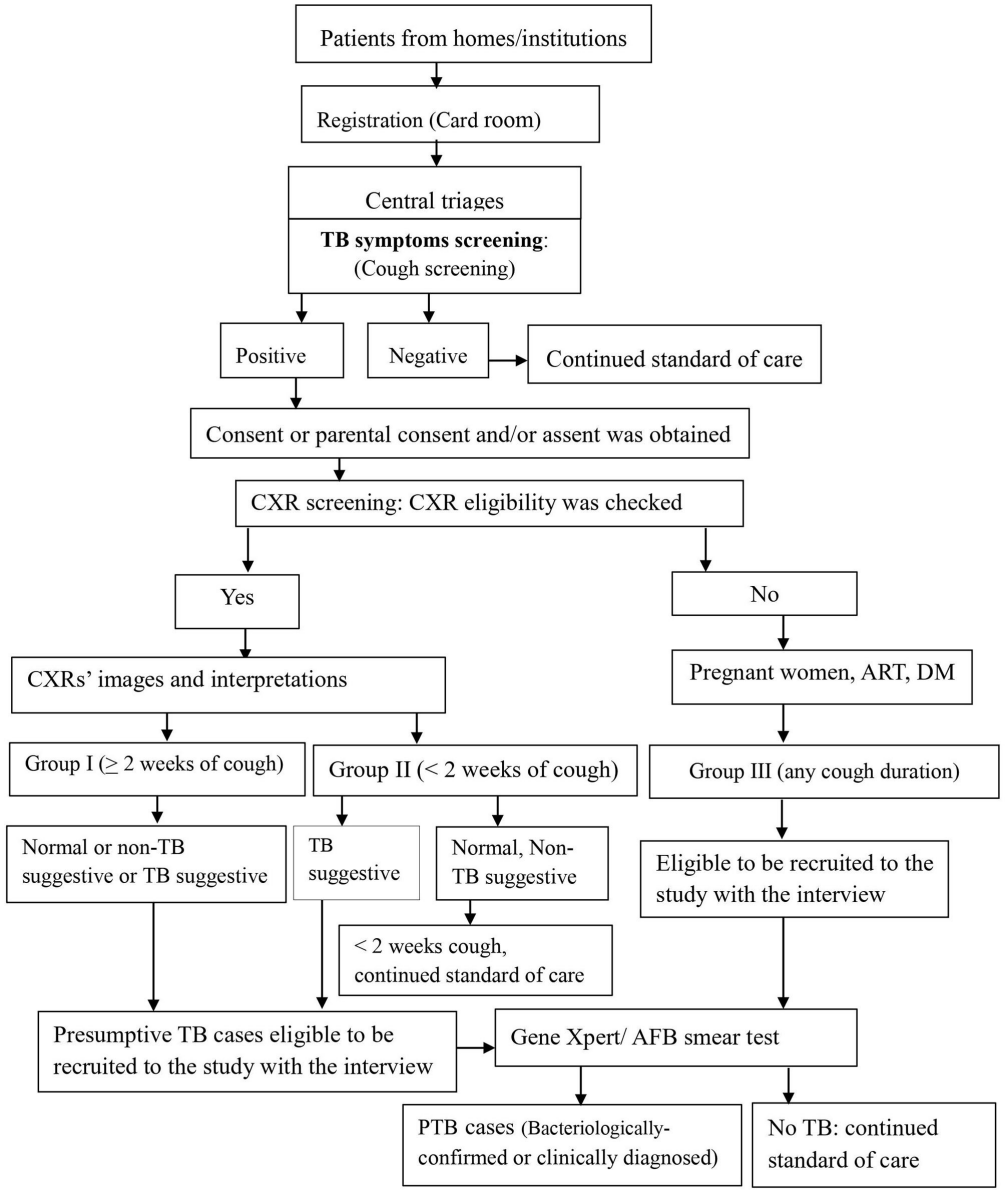
Screening, recruitment, and diagnosis workflow for PTB at health facilities, Ethiopia. RCH, Reproductive and child health; OPD, outpatients department; DM, Diabetic mellitus; CXR, Chest X-ray; PTB, pulmonary tuberculosis; AFB, Acid fast bacillus.

We screened for pulmonary TB using cough of any duration and/ or chest radiography among people who sought health care for any reason at study health facilities. Every day, for consecutive self–presenting patients, as study workflow, linking people were initiated at the registration (card rooms) of health facilities by stamping TB symptom(s)' screening stamp on patients' folders. When the patients' folders arrived at central triage, where patients visited and health care providers decided where/who participants would contact, health care providers screened the participants for TB symptom(s) by ticking “Yes” or “No” on patients' folders (Figure 1).

Screening steps were implemented in two ways: two sequential screening followed by one confirmatory test among chest X-ray group (symptom and CXR screenings) and one screening (symptom screening) followed by one confirmatory test among non-chest X-ray group (4, 22).

### Eligibility Criteria

Health care providers linked participants with cough of any duration to the data collectors, who requested the consent or parental consent based on the age per the Ethiopian and WHO ethics guidelines (23, 24). Step by step, when it was obtained, data collectors filled the eligibility and data collection tools (Figure 1). Further, study participants' eligibility was assessed for the chest X-ray (CXR) screening procedure and the enrollment into the study. Those TB patients who were on anti TB treatment before the start of the study and those who were diagnosed in other facilities and came to the study facilities for anti-TB treatment service were excluded.

### Study Participant Recruitment Procedures

After a written informed consent or parental consent was obtained and CXR eligibility was checked, patients received a coupon for free on-site or out-of-site CXR. For out-of-site CXR, transport was provided by the research project through an agreement entered with public health faculties that have X-ray machines. Posterior-anterior chest radiography was taken by experienced radiographers at the study hospitals or a public hospital for the patients from Melka Jebdu Health Center. CXR images were read by experienced radiologists who were blinded for the clinical or symptom screening results for study purposes. Radiologists put their interpretation as normal, TB suggestive, and what was observed on the image that later on recorded as a non-TB suggestive. For those who experienced cough of ≥2 weeks (group I), all three categories of CXR interpretations: normal, TB suggestive, and abnormal non-TB suggestive were eligible presumptive TB cases. For those who experienced cough of <2 weeks (group II), the TB suggestive TB CXR interpretation was eligible to recruit and investigate (Figure 1). For the non-CXR group (group III), the participant was identified, recruited, and investigated.

### Data Collection Process and Quality Control

Among participants, those who had cough screening positive were linked to the data collectors. The data were collected with a questionnaire that involved a structured questionnaire comprised of demographic variables, clinical symptoms, and medical history comprising previous TB and TB case contact in the last 2 years. Laboratory data and chest X-ray findings were recorded using forms. Data were collected by trained data collectors, and the collected data were checked daily. The principal investigator reviewed all questionnaires for completeness and accuracy when compared to the source documents.

We gave due emphasis to sputum laboratories: experienced laboratory technologists performed sputum tests as per national guidelines. We gave a thorough focus for the CXR quality control for both imaging performed by experienced radiographers and reading performed by experienced radiologists. We checked the CXR quality control by taking samples of TB suggestive and non-TB suggestive separately. A sample of CXR films (5% of those recruited with CXR) was randomly taken (*n* = 79) and read by another radiologist blindly to check the CXR reading reliability and calculated kappa agreement with 95% CI for PTB case identification. We obtained a 95% (95% CI: 87.5–98.6%) level of kappa agreement for TB suggestive abnormality identification.

### Sputum Laboratory Procedures

Sputum samples were collected in a falcon tube. Smear microscope processing, reading, and reporting were performed as per the Ethiopian national TB guidelines (9). Gene Xpert MTB/RIF assay processing was done according to manufacturer instructions (Cepheid, Sunnyvale, CA, United States). Laboratory technologists at the study facilities were performed the sputum samples as per the national TB guidelines.

### Statistical Analysis

Data were analyzed using Stata version 14.0 (Stata-Corp, College Station, Texas, USA). We assessed the screening algorithms with the yields of pulmonary TB cases diagnosed. We calculated the prevalence of pulmonary TB cases among the presumptive TB cases obtained from people screened for cough of any duration using symptom and/or chest X-ray screening at study health facilities. We assessed the predictor of TB cases using Poisson regression models with robust variance to compute Prevalence Ratio (PR) (25) with 95% confidence interval (CIs) and *p* < 0.05 was taken as statistically significant.

## Results

### Participants Characteristics

**Figure 2:**
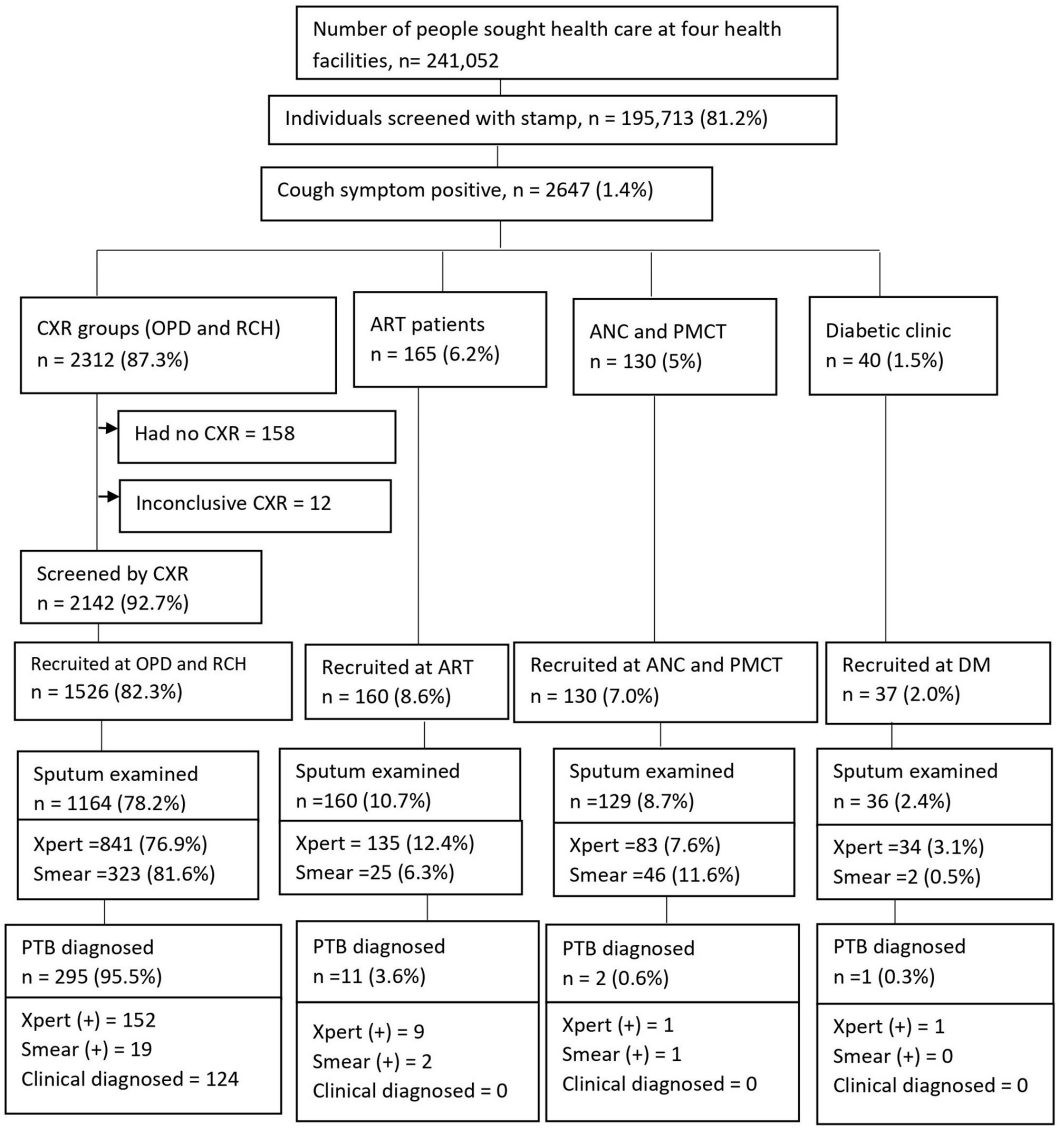
Screening yields for pulmonary tuberculosis cases at health facilities, Ethiopia. CXR, Chest X-ray; OPD, Outpatients department; RCH, Reproductive child health; ANC, Ante-natal care; PMCT, Prevent of mother to child transmission; DM, Diabetic mellitus; ART, Anti-retroviral therapy; PTB, pulmonary tuberculosis.

We screened 195,713 (81.2%) study participants for cough of any duration, out of a total of 241,052 people who sought health care at four health facilities found in Ethiopia. Of those screened, 2647 (1.4%) were cough symptom positive and agreed to participate in the study, of which 2312 (87.3%) were placed in the chest X-ray group and 335 (12.7%) were placed in the non-CXR group. Among the cough positive group, participants from outpatient departments (OPDs) comprised 2301/2647 (88.1%), and participants from reproductive and child health (RCH) comprised 11/2647 (0.42%) (Figure 2). A total of 1,853 presumptive TB cases fulfilled the eligibility criteria and were recruited into the study.

### Prevalence of Pulmonary TB Cases

Of 1,853 participants, the majority (1526, 82.3%) were recruited at OPDs and RCH. Sputum tests were performed for 1489 (80.4%) [1093 (73.4%) by GeneXpert and 396 (26.6%) by AFB smear microscopy] (Figure 2). The prevalence of pulmonary TB cases among people who sought health care with cough of any duration was as high as 309/1853 (16.7%); of which, 298 (95.5%) PTB cases were diagnosed at OPDs. Bacteriologically-confirmed pulmonary TB cases were 185 /309 (60%), whereas based on chest X-ray results, patients' histories, and clinical findings, clinically diagnosed PTB cases were 124/309 (40%) (Figure 2). Of 124 clinically diagnosed PTB cases, *Mycobacterium tuberculosis* (*Mtb*) was not detected by GeneXpert among 84 (67.7%), smear-negative among 26 (21%), and sputum tests were not performed for 14 (11.3%) and treated empirically.

Overall, 163 (53.1%) PTB cases were diagnosed among males The median age was 32 years (IQR: 22-50 years). By age, more PTB cases were diagnosed in the age group of 25–34 years, 96 (31.1%). More TB cases were diagnosed among participants who were rural (226, 73 %), married (199, 64.4%), and whose occupation was farming (105, 33.9%) (Table 1).

**Table 1:** Socio-demographic variables associated to pulmonary TB among presumptive TB cases at health facilities, Ethiopia.

**Characteristics**	**Pulmonary TB, *n* (%)**	**No TB, *n* (%)**	***p*-value**
**Gender**			
Female	146 (46.9)	821 (53.2)	0.045
Male	163 (53.1)	723 (46.8)	
**Age groups in years**			
<15	34 (11)	165 (10.7)	<0.0001
15–24	81 (26.2)	263 (17)	
25–34	97 (31.4)	311 (20.1)	
35–44	51 (16.5)	261 (16.9)	
45–54	29 (9.4)	231 (15)	
≥55	17 (5.5)	313 (20.3)	
**Residence**			
Rural	226 (73)	747 (48.4)	<0.0001
Urban	83 (27)	796 (51.6)	
**Marital status**			
Ever married	199 (64.4)	1,123 (73)	0.002
Never married	110 (35.6)	416 (27)	
**Educational status**			
Formal education	148 (47.9)	737 (47.8)	0.789
Never attended	146 (47.3)	716 (46.4)	
Informal education	15 (4.8)	90 (5.8)	
**Occupation**			
Farmer	105 (33.9)	341 (22.1)	<0.0001
Housewife	58 (18.8)	380 (24.6)	
Employed	31 (10)	269 (17.4)	
Business	26 (8.4)	110 (7.1)	
Student	49 (15.8)	185 (12)	
None	28 (9.1)	197 (12.7)	
Not applicable	12 (3.8)	62 (4)	

### Symptom Screening and Pulmonary TB Cases

Among 1,853 presumptive TB cases, we assessed TB symptoms as hemoptysis, fever, weight loss, and night sweats. Among PTB cases, 233 (75.4%) reported night sweats. By cough duration, the majority (264, 85.4%) of PTB cases diagnosed among the prolonged cough duration group (≥2 weeks). Among those who reported cough of <2 weeks for all groups (I to III), we found as high as 45 (15.6%) PTB cases (Table 2).

**Table 2:** Association between clinical symptoms, chest X-ray results, others, and pulmonary TB at health facilities, Ethiopia.

**Characteristics**	**Pulmonary TB, *n* (%)**	**No TB, *n* (%)**	***p*-value**
**Hemoptysis**			
Yes	58 (18.8)	179 (12)	0.001
No	251 (81.2)	1,365 (88)	
**Fever**			
Yes	145 (47)	595 (58.5)	0.006
No	164 (53)	949 (61.5)	
**Weight loss**			
Yes	94 (30)	330 (21)	0.001
No	215 (70)	1,211 (79)	
**Night sweats**			
Yes	233 (75.4)	821 (53.2)	<0.0001
No	76 (24.6)	723 (46.8)	
**Chest X-ray results**			
Normal	12 (4)	611 (49.6)	<0.0001
Non-TB suggestive	27 (9)	596 (48.4)	
TB suggestive	256 (87)	24 (2)	
**Algorithms**			
Non CXR group	14 (4.5)	313 (20.3)	<0.0001
CXR groups <2 weeks cough	44 (14.2)	5 (0.3)	
CXR groups ≥2 weeks cough	251 (81.2)	1,226 (79.4)	
**Cough duration**			
14 days and more	264 (85.4)	1,349 (87.4)	0.345
<14 days	45 (15.6)	195 (12.6)	
**Sought care for cough**			
Yes	300 (97)	1,391 (90.4)	<0.0001
No	9 (3)	148 (9.6)	
**Units of facilities**			
OPD and RCH	295 (95.5)	1,231 (79.7)	<0.0001
ART	11 (3.6)	149 (9.7)	
ANC and PMCT	2 (0.6)	127 (8.3)	
Diabetic clinics	1 (0.3)	36 (2.3)	

*OPD, Outpatients department; RCH, Reproductive child health; ANC, Ante-natal care; PMCT, Prevent of mother to child transmission; ART, Anti-retroviral therapy; CXR, Chest X-ray*.

### Chest X-Ray Screening and Pulmonary TB Cases

Of 1,526 (82.3%) presumptive TB cases that had CXR interpreted results, 623 (40.8%), 623 (40.3%), and 280 (18.4%) had normal chest X-ray, non-TB suggestive, and TB suggestive CXR results, respectively. The majority (256, 87%) of PTB cases were diagnosed among TB suggestive CXR. Among those who had cough ≥2 weeks or group I, with symptom screening, chest X-ray screening with any results, and Xpert /AFB smear, we found 251/309 (81.2%) PTB cases. Among those who had cough of <2 weeks or group II, with symptom screening, chest X-ray screening with TB-suggestive results, and Xpert / AFB smear, we found 44/309 (14.2%) PTB cases. Among those who had cough of any duration or group III, and Xpert/AFB smear among pregnant women, ART, and diabetic patients, we found 14/309 (4.5%) PTB cases (Table 2).

By cough duration, a total number of participants with cough <2 weeks and ≥2 weeks that screened by CXR were 661/2142 (30.8%) and 1481/2142 (69.2%), respectively. Among those participants with cough <2 weeks and ≥2 weeks, TB suggestive CXR results were 49/661 (7.4%) and 232/1481 (15.7%), respectively.

### PTB by Screening Algorithm and Sputum Tests Performed (Xpert/Smear)

PTB cases diagnosed by the algorithm of symptom screening, CXR screening, and Xpert were 152/236 (64.4%), with Xpert negative among 84/236 (35.6%), but clinically diagnosed based on CXR results, patients' histories, and clinical findings (Table 3).

**Table 3:** Pulmonary TB case finding algorithms of cough and/or chest X-ray screening then followed by Xpert/smear microscopy at health facilities, Ethiopia.

**Screening algorithms and laboratory performed**	**Xpert/smear positive**	**Xpert/smear negative but clinical diagnosed as PTB cases**	**Total PTB cases**
Cough screening followed by CXR screening, then GeneXpert	152	84	236
Cough screening followed by CXR screening, then AFB smear microscopy	19	26	45
Cough screening followed by GeneXpert	11	0	11
Cough screening followed by AFB smear microscopy	3	0	3
Diagnosed based on only symptoms and CXR (empirical treatment)	-	-	14
Total PTB cases	185	124	309

*CXR, Chest X-ray; PTB, pulmonary tuberculosis*.

### Predictors of Pulmonary Tuberculosis Among Presumptive TB Cases

In multivariable analysis, among people screened and tested for TB, age group was a statistically significant predictor for the diagnoses of TB. For example, as the age group from 15 to 24 years and 25 to 34 years were (aPR = 2.2, 95% CI: 1.5–3.2) and (aPR = 2.0, 95% CI: 1.3–2.8) times as likely to be diagnosed with PTB as age group ≥55 years, respectively.

From clinical symptoms of TB, those who experienced a history of weight loss were (aPR = 1.2, 95% CI: 1.1–1.3) times more likely to be diagnosed with PTB than individuals who had no history of weight loss. Those who had a history of contact with TB cases in the last 2 years were 1.2 (95 % CI: 1.0–1.4) times likely to be diagnosed with PTB than those who had no contact. Those individuals with TB suggestive chest X-ray findings were 41.1 (95% CI: 23.2–72.8) times likely to be diagnosed with TB than those who had normal chest X-ray results (Table 4).

**Table 4:** Factor associated to pulmonary TB among presumptive TB cases at health facilities, Ethiopia.

**Characteristics**	**cPR (95% CI)**	**aPR (95% CI)**
**Gender**		
Female	1	1
Male	1.23 (1.0–1.5)^*^	1.0 (0.9–1.1)
**Age groups in years**		
0–14	3.3 (1.9–5.8)^***^	1.8 (1.2–2.6)^**^
15–24	4.6 (2.7–7.6)^***^	2.2 (1.5–3.2)^***^
25–34	4.5 (2.7–7.5)^***^	2.0 (1.3–2.8)^***^
35–44	3.1 (1.8–5.3)^***^	1.7 (1.2–2.5)^**^
45–54	2.1 (1.2–3.8)^**^	1.7 (1.2–2.5)^**^
≥55	1	1
**Residence**		
Rural	2.35 (1.87–2.96)^***^	1.3 (1.1–1.5)^***^
Urban	1	1
**TB case contact**		
Yes	1.6 (1.2–2.1)^*^	1.2 (1.0–1.4)^*^
No	1	1
**Bloody sputum**		
Yes	1.5 (1.2–2.)^***^	1.1 (0.9–1.3)
No	1	1
**Fever**		
Yes	1.3 (1.1–1.6)^***^	1.1 (0.9–1.2)
No	1	1
**Weight loss**		
Yes	1.4 (1.2–1.8)^***^	1.2 (1.0–1.3)^*^
No	1	1
**Night sweats**		
Yes	2.3 (1.8–2.9)^***^	1.1 (0.9–1.2)
No	1	1
**Sought care for cough**		
Yes	3.1 (1.6–5.8)^**^	1.1 (0.6–1.6)
No	1	1
**Chest X-ray results**		
Normal	1	1
Non TB suggestive	2.2 (1.2–4.4)^*^	2.4 (1.2–4.7)^*^
TB suggestive	47.3 (26.8–82.6)^***^	41.1 (23.2–72.8)^***^

*Cpr, Crude prevalence ratio; aPR, Adjusted prevalence ratio; PTB, pulmonary tuberculosis*.

*Statistical significance*:

* *p < 0.05*,

** *p < 0.01*,

*** *p < 0.001*.

## Discussion

We studied the prevalence of PTB cases by screening for cough of any duration and/or chest X-ray among people who sought health care for any reason at health facilities. To this end, we applied three different algorithms to detect PTB cases. The first algorithm is as follows: cough of ≥2 weeks followed by chest X-ray screening with any results that followed with Xpert /AFB smear microscopy (group I), and with this algorithm we found 81.2% PTB cases. The second algorithm was: cough of <2 weeks followed by chest X-ray screening with TB-suggestive results followed with Xpert /smear microscopy (group II), and with this algorithm we found 14.2% PTB cases. The third algorithm was: cough of any duration followed by Xpert/smear microscopy (group III) among pregnant women, ART and diabetic patients, and with this algorithm we found 4.5% PTB cases. Overall for all groups I to III, we found that 1 in 4 [309/1853 (16.7%)] PTB cases were diagnosed, with significantly higher among the working-age group of 25–34 years (31.1%).

The prevalence of PTB cases diagnosed among people with cough of any duration at health facilities was 16.7%, which was higher than the number of PTB cases diagnosed using passive screening as studies at Addis Ababa, Ethiopia, which revealed 15.1% (26) and 13.5% (27). Diagnosing more PTB cases in this study than in other studies might be we used sensitive CXR screening and cough of any duration rather than follow the national TB program (NTP) that uses cough of ≥2 weeks except for HIV/AIDs patients with cough of any duration (9). However, it was lower than studies in Debre Markos, northwest Ethiopia, which reported 23.2% (28), and in Nigeria, which reported 37.7% (29). The variation could be due to the difference in the study populations, times and methods. TB was common among the working-age group, which is similar to a study in Bahir Dar, Ethiopia (30).

Among target groups, we did screened a high number of PTB cases (95.5%) that were diagnosed among people who attended OPDs, which was higher than studies from Ghana (79.7%) (31) and Nigeria (80%) (16). These differences might be due to the study populations, screening and diagnosis methods used, and the settings of the screening. Besides, the current study shows 9 (3%) cases were diagnosed among those who sought care for non-respiratory concerns (cough). This finding was comparable to a finding of TB cases among those missed by screening in south Ethiopia (3.4%) among health facilities attendants of OPDs (32) and among impatiens (3.3%) in central Ethiopia (33), but lower than a study from South Africa (5%) (34). The variation could be due to the differences in study populations, settings, and algorithms.

Using the algorithms of cough screening followed by chest X-ray screening (group I and group II) followed by GeneXpert sputum yielded 64.4% of PTB cases (Table 3), which corroborate the recommendation of using Xpert as an initial diagnostic test at the point of care (35, 36). The algorithms also required less change to be setting-specific (22), so if a given facility has the X-ray machine and radiologist, it can be integrated into the routine standard of care. As per the World Health Organization recommendation, CXR screening is a good choice at health facilities that reduces the costs and logistical challenges when compared with active case finding in communities (37). The NTP of Ethiopia TB guidelines have been indicated for the use of CXR at early for triaging, screening, and assisting in the diagnosis of TB among those of clinical high-risk groups that have sought care for any complaint (9).

Furthermore, we applied the screening with a reduced cough duration (<2 weeks) combined with CXR screening criterion of TB suggestive. It was useful in reducing a number of sputum examination (Figure 1), and among the CXR group we found 14.2% of TB cases that could have been missed if we had used the common TB screening of >2 weeks cough as a criterion for sputum examination. For example, in the current study, 28.6% (95% CI: 26.7–30.6%) tests were reduced for the algorithm of less <2 weeks among those who had normal and non-TB suggestive CXR results. The reduced Xpert tests were lower than a study from Nepal that reported 31-60% (38). The reason for this difference might be due to study population, algorithms, and criteria used for CXR reading, and they performed sputum for those with any abnormal CXR results, age 15 years or older, and cough more than 14 days. Using a reduced cough duration with CXR screening of criteria of TB suggestive could help in detecting TB cases early which has additional health value for individuals by reducing the severity of disease consequences and reducing transmission in the community, which are the two ultimate goals of screening (39, 40).

Using the algorithm of cough of any duration followed by sputum tests (group III) we found 4.5% of PTB cases. This algorithm was feasible to implement at health facilities including at primary health care level such as health centers, but only few of these facilities have GeneXpert machines in Ethiopia (41), the sputum tests depends on the smear microscopy that has been reported with a less sensitivity than GeneXpert to detect TB (35) and delayed the TB patients (42). Using cough screening is useful in the era of COVID-19 when the TB services were impacted (43) and the knowledge of the community for COVID-19 was inadequate (44).

We found that a high number of PTB cases were diagnosed with smear/bacteriological negative that were 40% based on CXR results, patients' histories, and clinical findings as per NTP (9). A study from southwest Ethiopia, by clinical algorithm, identified a high number of confirmed TB among smear-negative patients using cultures (45). In this case, using CXR screening at an early stage by paying its cost for the health facilities or making it free for the patients, as with other TB tests such as GeneXpert, could help in controlling losses to follow-up among smear/ bacteriological negative TB cases including those who could not be returned to health facility again when the appointment was given for them (46–48) and for the fear of CXR and transportation costs (6). We recommended that the NTP reconsider the cost of CXR for TB patients. Besides, TB suggestive CXR findings were a strong indicator to be diagnosed with PTB cases [aPR = 41.1, (95% CI: 23.2–72.8)]. We implemented sequential screening algorithms that symptom followed by CXR that if TB suggestive among those with cough of any duration for those attended OPD and RCH. This finding was similar to that found by a study from Vietnam (49).

We observed that using cough of any duration screening followed by CXR followed by Xpert/smear among eligible individuals might increase the feasibility for health facilities at different units including CXR and laboratory, which could happen when any symptom (that with low positive predictive value) was used in a study approached 40% of daily visits (50). Further, in this study, using similar entry points by stamping TB symptoms' stamp on all health seeking attendants' medical folders were used, rather than selective screening, in order to approach both with or without symptoms compatible with TB to control missed opportunities (39). This is helpful to control the probability of approaching more participants who were severely ill or with respiratory symptoms than others by data collectors (51). The possible limitation of cough screening might be missed in a few cases without cough or asymptomatic individuals.

Our study has the following strengths: first, in increasing the number of people screened for cough of any duration than screened for cough of ≥2 weeks among people who sought health care for any reason. Second, it covered different target groups at different health facilities (both hospitals and health center) that were found in rural and urban settings. Third, using more sensitive screening algorithm (cough of any duration and/or chest X-ray) that is used for the diagnosis of PTB including clinical diagnosis as per the NTP recommendation to detect and treat bacteriologically missed TB in real settings. Our study is one of the studies that investigated tuberculosis through screening with different algorithms (9). However, it was not without limitations: first, this study demonstrated different numbers of PTB cases using different screening and diagnostic algorithms, to compare between the algorithms, it would be better if sputum were performed by both GeneXpert and AFB smear microscopy at the same time, but we used one or the other. Second, we did not perform sputum culture as it has not been used in routine standard of care for TB diagnosis in Ethiopia (9). This might have reduced the number of PTB cases diagnosed with bacteriologically-confirmed, but we used a sensitive screening of chest radiography, and the sensitivity of GeneXpert was well-documented. With this, it can be used for similar settings at health facilities.

## Conclusion

The prevalence of confirmed PTB among routine outpatients was high, and this included those with a low duration of cough who can serve as a source of infection. Screening all patients at outpatient departments who passively report any cough irrespective of duration is important to increase TB case finding and reduce TB transmission and mortality.

## Data Availability Statement

The data used to support the conclusion of this article are included in the article. Datasets used to analyze this article is available and can be shared upon reasonable request from the corresponding author.

## Ethics Statement

The studies involving human participants were reviewed and approved by Institutional Review Board (IRB) of the College of Health Sciences, Addis Ababa University (Ref. No. AAUMF 03-008) and Haramaya University, College of Health and Medical Sciences, Institutional Health Research Ethics Review Committee (IHRERC) (Ref. No. IHRERC/004/2019). Written informed consent to participate in this study was provided by the participants' legal guardian/next of kin.

## Author Contributions

HM conceived the concept, designed the study protocol, coordinated and analyzed the data, and wrote and revised the manuscript. LO, KR, EN, TM, and AF supervised and reviewed the protocol and manuscript. TA reviewed the manuscript and coordination. NM participated in data supervision and reviewed the manuscript. GY reviewed the protocol, supervised, reviewed the manuscript, and worked on administration and financial acquisition. All authors approved the final version for publication.

## Funding

This study was helped by the EXIT-TB project which is part of the European & Developing Countries Clinical Trials Partnership 2 (EDCTP2) program, supported by the European Union (grant number CSA2016S-1608). The funder had no role in the conception, data collection, analysis, and writeup.

## Conflict of Interest

The authors declare that the research was conducted in the absence of any commercial or financial relationships that could be construed as a potential conflict of interest.

## Publisher's Note

All claims expressed in this article are solely those of the authors and do not necessarily represent those of their affiliated organizations, or those of the publisher, the editors and the reviewers. Any product that may be evaluated in this article, or claim that may be made by its manufacturer, is not guaranteed or endorsed by the publisher.

## Abbreviations

ANC: Ante-natal care
ART: antiretroviral therapy
CXR: Chest X-ray
DM: Diabetic Mellitus
FMoH: Federal Ministry of Health
HIV: Human immunodeficiency virus
IQR: Interquartile range
NTP: National TB program
OPD: Outpatient Department
PMCT: Prevention of mother to child transmission
PID: Patient identification number
PTB: Pulmonary tuberculosis
RCH: Reproductive Child Health
TB: Tuberculosis
WHO: World Health Organization.
